# Antioxidant and Skin Whitening Activities of Sub- and Super-Critical Water Treated Rutin

**DOI:** 10.3390/molecules27175441

**Published:** 2022-08-25

**Authors:** Yeon Jae Jo, Dan Hee Yoo, In Chul Lee, Junsoo Lee, Heon Sang Jeong

**Affiliations:** 1 Department of Food Science and Biotechnology, Chungbuk National University, Cheongju 28644, Korea; 2 College of Fusion and Convergence, Seowon University, Cheongju 28674, Korea; 3 Department of Bio-Cosmetic Science, Seowon University, Cheongju 28674, Korea

**Keywords:** supercritical water, subcritical water, rutin, skin whitening, antioxidant

## Abstract

We focused on the functional components, antioxidant activity, skin-whitening, and anti-wrinkle properties of subcritical and supercritical water (SCW)-treated rutin. Rutin treatments were performed at the following temperature and pressure conditions: 200 °C/15 bar, 300 °C/100 bar, and 400 °C/250 bar. ABTS and DPPH radical scavenging activities and reducing power presented their highest values (1193.72 mg AAE/g, 728.73 mg AAE/g, and 0.65, respectively) at 300 °C/100 bar. The tyrosinase inhibitory activity of SCW-treated rutin was 21.72–60.05% at 1 mg/mL. The ethyl acetate fraction showed 14.91% melanin inhibitory activity at a concentration of 10 µg/mL compared to the α-MSH treatment group. The protein expression inhibition rates of MITF, tyrosinase, TRP-1, and TRP-2 in the ethyl acetate fractions were 14.05%, 72%, 93.05%, and 53.44%, respectively, at a concentration of 10 µg/mL, compared to the control. These results indicate that SCW treatment could be used to develop cosmetic materials and functional food with physiological activity, and that SCW-treated rutin can be used as a skin-whitening cosmetic material.

## 1. Introduction

Supercritical water (SCW) refers to water at pressure and temperature above the critical point (218 bar/374 °C), presenting similar properties to solids, liquids, and gases [1,2]. SCW has a very high ionic strength. Its hydrogen ion concentration is increased by more than 30 times, whereas its pH is reduced to 3. Such properties provide an environment similar to the conditions needed for acid-catalyzed reactions, while it can also dissolve organic compounds, such as benzene [3]. Therefore, SCW has various applications, such as in the production of fine particles, decomposition reactions of incombustible substances, and synthetic, radical, and ionic reactions [4,5,6].

Rutin is a glycoside in which the flavonoid quercetin and the disaccharide ruinose (α-l-rhamnopyranosyl-(1-6)-β-d-glucopyranose) are combined. Rutin presents various physiological characteristics, such as antioxidant, anti-diabetic, anti-cancer, and anti-aging. Rutin is known to increase its activity when converted to an aglycone, quercetin, and deglycosylation occurs by various methods, such as chemical treatment, acid treatment, and heat treatment [7]. It can prevent bleeding by strengthening capillaries and regulating blood pressure. Furthermore, it can inhibit cerebral hemorrhage, hemorrhagic diseases, and radiation disorders [8,9,10]. Hesperidin, quercetin, and chalcone are named vitamin P and are used as food additives in the food industry. In the cosmetic industry, vitamin C and citrin are used as ingredients for skin improvement and capillary expansion products.

Melanin is a group of pigments that is widely distributed in living organisms and synthesized by melanocytes in the epidermal layer. It darkens the skin color due to pigmentation or substances produced to inhibit cell damage. Tyrosinase is the primary enzyme concerned in melanin biosynthesis. In the past, the development of whitening materials focused on tyrosinase inhibitory activity, but as the autooxidation inhibitory activity has been recently highlighted, research on various mechanisms for inhibiting melanin synthesis has been actively conducted [11].

The effect of high-temperature and -pressure treatments on the biological activity of various agro-foods have been the focus of several studies, verifying that antioxidant, anti-inflammatory, and anti-cancer properties of the products were enhanced after such treatments. However, these studies have only focused on the low subcritical water region below 150 °C [12,13,14], whereas higher-temperature SCW treatment has the potential to create new materials and enhance the biological activities. Therefore, the purpose of this study was to evaluate the antioxidant and skin anti-aging activities of SCW-treated rutin. We presented the potential of the SCW treatment technology for developing materials with enhanced physiological activities and verified the potential of rutin as a cosmetic material and functional food.

## 2. Results and Discussion

### 2.1. Rutin and Quercetin Content

**Figure 1:**
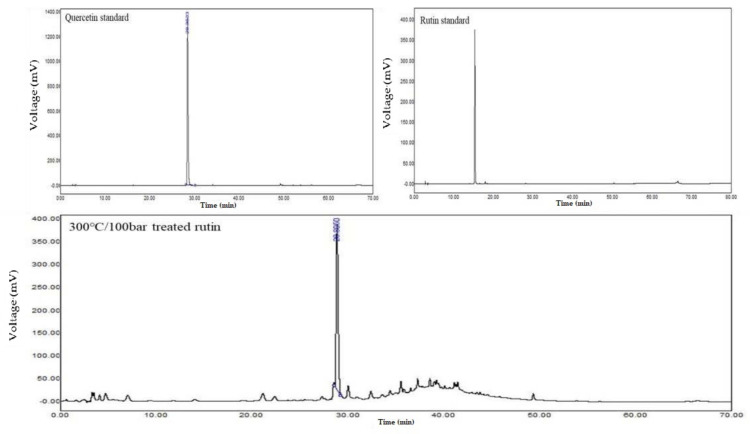
Chromatogram of rutin and quercetin standard and 300 °C/100 bar treated rutin sample.

The rutin and quercetin contents of the SCW-treated rutin are shown in Table 1, and the chromatogram of rutin and quercetin standard and 300 °C/100 bar treated rutin sample is shown in Figure 1. Rutin was not present in all samples, whereas the quercetin content was 1.40 mg/g at 400 °C/250 bar and 381.38 mg/g at 200 °C/15 bar. According to Vetrova et al. [15], when rutin was treated with subcritical water, the quercetin content increased with increasing the temperature up to 200 °C but decreased at temperatures above 200 °C. The roasting procedure of noni leaves produced flavonol aglycones from glycosides, which could lead to enhanced bioactivity [16,17]. In aqueous solutions, all flavonoids are decomposed when heated at temperatures above 100 °C. This finding is similar to our results and is due to the breakdown or change in their structure in such a way that the HPLC/DAD signal of the primary structure can no longer be detected [18].

### 2.2. ABTS and DPPH Radical Scavenging Activity

The radical scavenging activity of the SCW-treated rutin is presented in Table 1. The ABTS assay ranged from 465.97 mg AAE/g in raw samples to 1193.72 mg AAE/g in samples at 300 °C/100 bar. The DPPH assay ranged from 656.08 to 728.73 mg AAE/g. When rutin was decomposed into quercetin, the antioxidant activity increased, so it was expected that the antioxidant activity of rutin treated at 200 °C/100 bar would be the highest. However, both ABTS and DPPH assays were measured to be high at 300 °C/100 bar treatment. These results suggest that other materials produced in addition to quercetin exhibit high antioxidant activity when rutin is treated with subcritical water and supercritical water. ABTS and DPPH assay were higher when rutin was treated at 300 °C/100 bar. The antioxidant activity can be increased using subcritical water treatment. According to Jo et al. [19], the antioxidant compound content in golden oyster mushrooms, such as phenolic compounds and β-glucan, increases with the increase in temperature and pressure of the subcritical water. This finding is in agreement with our results.

### 2.3. Reducing Power

The antioxidant capacity of a particular compound is related to its reducing power value. Therefore, reducing power can serve as an important indicator of the potential antioxidant activity [20]. The reducing power is confirmed by measuring the reduction of the Fe^3+^ of the Fe^3+^/ferricyanide complexes to the form of ferrous (Fe^2+^) [19]. Table 1 shows the effect of SCW-treated rutin on the reducing power: The reducing power was in the range of 0.31–0.65, with the 300 °C/100 bar SCW-treated rutin being the most active (0.65), and the non-treated rutin presenting the lowest value (0.31). According to Kim et al. [21], the SCW extract of citrus pomace showed the highest reducing power (0.311) at 200 °C for 60 min, and chaga mushrooms extracted with subcritical water showed higher reducing power compared to the others [22]. These results indicate that the antioxidant activity of rutin, such as reducing power, can be increased by high-temperature and high-pressure treatment.

### 2.4. Tyrosinase Inhibitory Activity

**Figure 2:**
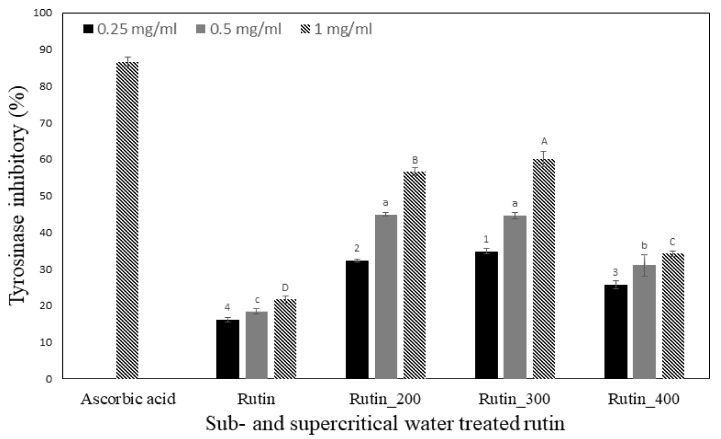
Tyrosinase inhibitory activity of rutin treated by different pressure and temperature. Data are expressed as mean ± SD. Different letters and numbers in the same items indicate a significant difference among different samples by Duncan’s range test (*p* < 0.05).

Under normal conditions, melanin increases the skin’s resistance to irritation caused by ultraviolet rays, but excessive biosynthesis causes pigmentation and skin damage, such as spots, freckles, and age spots. The inhibition of tyrosinase activity causes skin whitening, and research on physiologically active substances that effectively inhibit tyrosinase is therefore very important in the cosmetic industry [23]. We examined the possibility of using SCW-treated rutin as a whitening cosmetic material, and its tyrosinase inhibitory activity is shown in Figure 2. Ascorbic acid was used as the positive control and measured an inhibitory rate of 86.59% at a concentration of 1 mg/mL, whereas the values obtained from 1 mg/mL sub- and SCW-treated rutin were 21.72–60.05%. Among them, SCW-treated rutin at 300 °C/100 bar measured the highest inhibitory activity (60.05% at 1 mg/mL). Our results are in agreement with Koyu et al. [24], who reported the tyrosinase inhibitory activity of subcritical water extracts of Morus nigra fruits and found that the tyrosinase inhibitory activity increased as the subcritical water extraction temperatures increased.

### 2.5. Solvent Fraction

**Figure 3:**
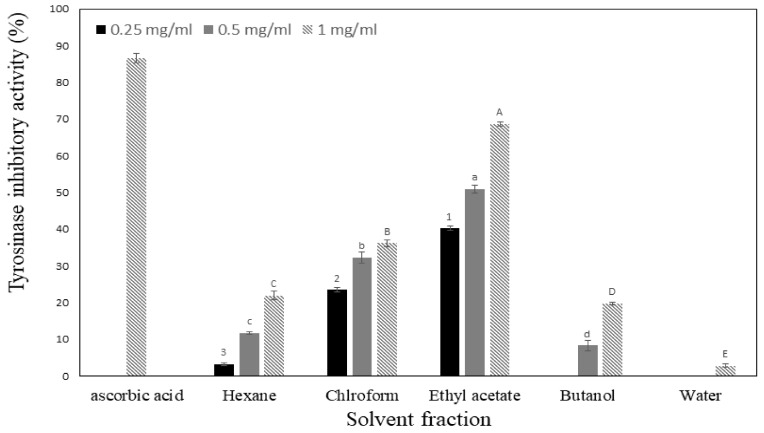
Tyrosinase inhibitory activity of solvent fraction for 300 °C and 100 bar treated rutin. Data are expressed as mean ± SD. Different letters and numbers in the same items indicate a significant difference among different samples by Duncan’s range test (*p* < 0.05).

The antioxidant and skin-whitening activities of SCW-treated rutin were studied. Rutin treated at 200 °C/15 bar showed the highest quercetin content, whereas rutin treated at 300 °C/100 bar showed the highest antioxidant and skin-whitening activities. Thus, we performed solvent fractionation of rutin treated at 300 °C/100 bar for separation and purification of antioxidant and skin anti-aging active substances. The quercetin content in the solvent fractions of rutin treated at 300 °C/100 bar is shown in Table 2. The quercetin content range was 0–248.48 mg/g. The highest value was observed in the ethyl acetate fraction, whereas quercetin was not detected in the butanol and water fractions. The ABTS assay was in the range of 0–1358.64 mg AAE/g, with the ethyl acetate fraction presenting the highest value. The DPPH radical scavenging activity presented values similar to those of ABTS, the range was 0–1410.73 mg AAE/g. Both ABTS and DPPH radical scavenging activities presented their highest values in the ethyl acetate fraction, followed by the hexane and chloroform fractions, whereas small or zero amounts were detected in the butanol and water fractions. The reducing power ranged from 0 in the water fraction to 0.77 in the ethyl acetate fraction. The tyrosinase inhibitory activity is shown in Figure 3. At a concentration of 1 mg/mL, it presented its lowest value (2.83%) in the water fraction and its highest value (68.62%) in the ethyl acetate fraction.

### 2.6. Cytotoxicity

**Figure 4:**
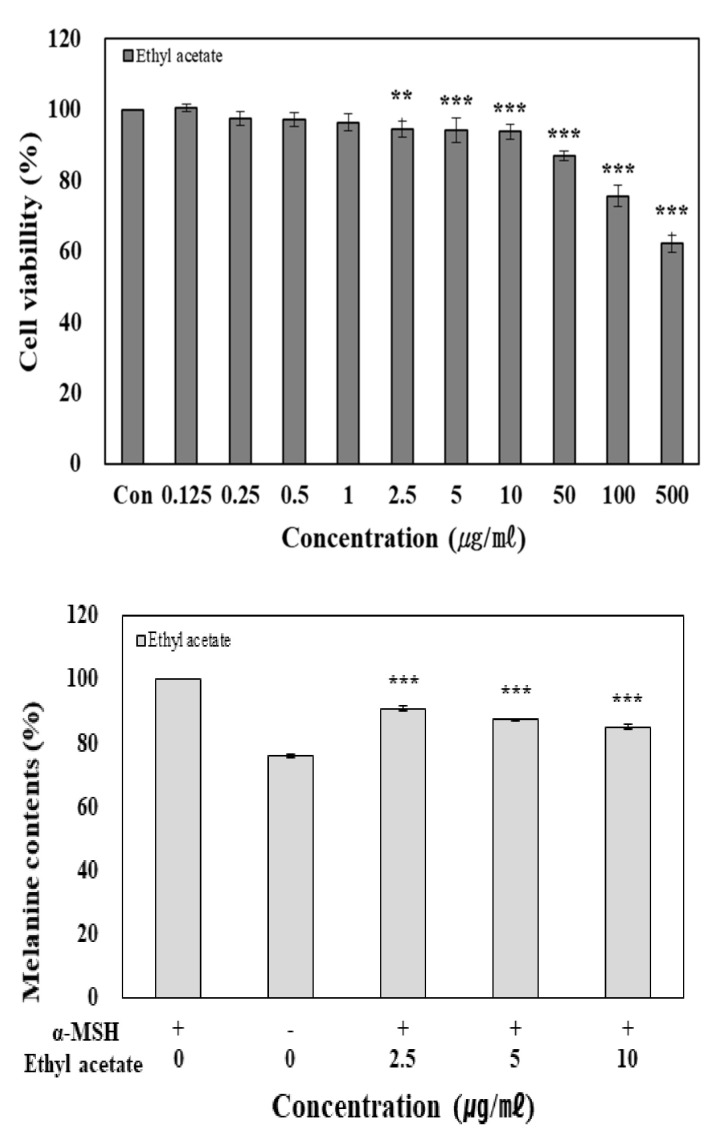
Cell viability and inhibition melanin synthesis of ethyl acetate fraction on B16F10 cells. B16F10 cells (1 × 10^4^ cells/well) were treated with various concentrations (1, 2.5, 5, 10, 50, 100, and 500 µg/mL) of extracts prior to the determination of cellular viability through MTT assay and were treated with ethyl acetate and α-MSH. ** *p* < 0.01, *** *p* < 0.001; significant difference compared to control.

Since the highest antioxidant and skin-whitening activity of solvent fractions of rutin treated at 300 °C/100 bar were observed in the ethyl acetate fractions, cell experiments were performed using this fraction. Cell viability measurements were performed using a colorimetric method, where a purple color is developed when mitochondrial dehydrogenase and MTT tetrazolium react during cell metabolism to form MTT formazan, which is then dissolved in DMSO [25]. The viability of B16F10 melanoma cells exposed to the ethyl acetate fraction was measured using the MTT assay, and the results are shown in Figure 4. The ethyl acetate fraction showed a cell survival rate of 93.83% at 10 μg/mL. At higher concentrations, the cell survival rate was less than 87.00%. Therefore, experiments using B16F10 melanoma cells were conducted at concentrations of 2.5, 5, and 10 μg/mL, in which more than 90% of the cells were viable.

### 2.7. Inhibition of Melanin Synthesis

Melanin is synthesized from the melanocytes present in the basal layer of the epidermis and transferred to keratinocytes and is one of the main factors determining the skin color of humans [26]. When melanin production in cells increases as a defense mechanism against environmental conditions, such as excessive UV exposure, it triggers pigmentation to cause skin aging and, in severe cases, melanoma [27]. In this study, the effects of the ethyl acetate fraction on melanogenesis were investigated. B16F10 cells were treated with 1 µg/mL of α-melanocyte-stimulating hormone (MSH) to induce melanogenesis, and then 2.5, 5, and 10 µg/mL of the ethyl acetate fractions were added. As shown in Figure 4, the ethyl acetate fraction showed 14.91% inhibitory activity at the final concentration of 10 µg/mL compared with that of the α-MSH treatment group.

### 2.8. MITF, TRP-1, TRP-2, and Tyrosinase Protein Expression Rate

**Figure 5:**
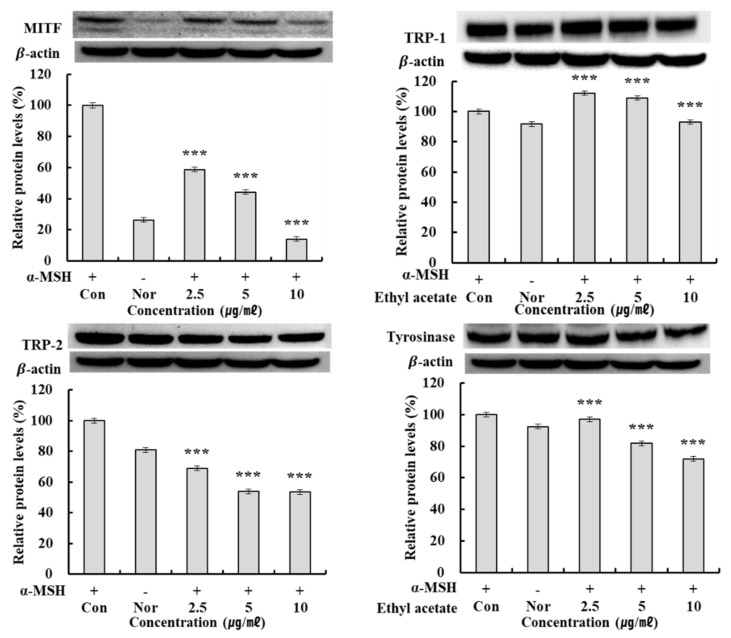
MITF, TRP-1, TRP-2, tyrosinase protein expression rate of ethyl acetate fraction on melanoma cells. B16F10 cells were incubated for 24 h in DMEM containing 10% FBS and then treated with various concentrations (2.5, 5, and 10 µg/mL) of solvent fraction for 24 h. MITF, Tyrosinase, TRP-1, TRP-2 protein level was determined by western blot. Con: control, in B16F10 cells treated with α-MSH, Nor: Normal, in B16F10 cells not treated with α-MSH. Results are means ± SD of triplicate data. *** *p* < 0.001; significant difference compared to control.

The production of melanin in the living body mainly occurs through an oxidative reaction involving the amino acid tyrosine. The enzymes related to this reaction include tyrosinase, TRP-1, and TRP-2. These enzymes convert DOPA to DOPA-quinone and, through non-enzymatic and auto-oxidation processes, to DOPA-chrome. These proteins are regulated by the MITF, which is an important transcriptional regulator of melanin synthesis and can inhibit melanin production [28]. In this study, the activity of MITF, tyrosinase, TRP-1, and TRP-2 factors on protein expression was evaluated to confirm the whitening effect of the ethyl acetate fraction. Figure 5 shows the MITF, tyrosinase, TRP-1, and TRP-2 protein expression after the treatment of melanoma cells. β-actin was used as the positive control. The protein expression of MITF, tyrosinase, TRP-1, and TRP-2 compared to the alpha-MSH processing group showed expression suppression activity of 14.05%, 72%, 93.05%, and 53.44%, respectively, at 10 μg/mL.

## 3. Materials and Methods

### 3.1. Materials

Rutin, quercetin, 2,2-azino-bis(3-ethylbenzothiazoline-6-sulphonic acid) diammonium salt (ABTS), 1,1-diphenyl-2-picrylhydrazyl(DPPH), hemoglobin, tyrosinase, elastase, collagenase, 3-[4,5-dimethylthiazol]-2-yl]-2,5-diphenyl-tetrazolium bromide (MTT), dimethyl sulfoxide (DMSO), α-MSH, radio-immunoprecipitation assay buffer (RIPA buffer), protease and phosphatase single-use inhibitor cocktail 100X, and the bicinchoninic acid (BCA) kit were purchased from Sigma Chemicals Co (St. Louis, MO, USA).

### 3.2. Subcritical and SCW Treatment

Subcritical and SCW treatments of rutin were performed using a SCW reactor (Ilsin autoclave Inc., Daejeon, Korea), reaching temperatures up to 400 °C with maximum pressure up to 300 bar. The pressure of the SCW reactor was set depending on the amount and temperature of water placed in the inner container. Three temperature/pressure conditions were tested: 200 °C/15 bar, 300 °C/100 bar (subcritical condition), and 400 °C/250 bar (supercritical condition). After reaching the desired temperature, the treatment was conducted for 30 min. A group of untreated samples was used as the control group. In the experiment, 0.3 g of rutin was suspended in 10 mL of distilled water and placed in the inner container, which was then placed in the outer container together with a certain amount of water, and the outer container was completely sealed. Rutin was treated with subcritical and SCW under each condition. The processed rutin was filtered using a 0.45 μm syringe filter (Millipore, Billerica, MA, USA), and solvent was removed at 40 °C in a rotary evaporator (EYELAN-1000, EYELA, Tokyo, Japan) and then freeze-dried (FD5508, Ilshin Lab Co., Ltd., Seoul, Korea). The dried sample was used and stored at −80 °C.

### 3.3. Solvent Fractionation

For solvent fractionation, we used 300 °C/100 bar treated rutin, which had the highest antioxidant and skin whitening activities. After SCW treatment, the sample was suspended in distilled water, and solvent fractionation was sequentially performed using 300 mL (x3) of each solvent in the following order: N-hexane, chloroform, ethyl acetate, butanol, and water [29,30]. Each solvent layer was dried using a rotary vacuum concentrator (EYELA N-1000, Tokyo Rikakikai Co., Tokyo, Japan), and a powder was obtained which was stored at −20 °C until further use. Analysis of the antioxidant and skin anti-aging properties was performed using each solvent fraction powder at a constant concentration.

### 3.4. Measurement of Rutin and Quercetin Content

Rutin and quercetin content analyses were conducted using slightly modified methods of Seo et al. and Jung et al. [31,32]. The SCW-treated rutin and solvent fractions were dissolved in HPLC-grade methanol, filtered using a 0.45 μm syringe filter (Millipore, Billerica, MA, USA), and analyzed by an HPLC system (Jasco System, Tokyo, Japan) equipped with an ODS column (Mightysil RP-18 GP 5 μm, 4.6 × 250 mm, Kanto Chemical, Tokyo, Japan). The method involved the following conditions. The mobile phase consisted of (A) 0.1% formic acid in acetonitrile and (B) 0.5% formic acid in water. The gradient was set at 0–2 min 8:92 (%, *v*/*v*), 2–27 min 10:90, 27–50 min 30:70, 50–51 min 90:10, 51–60 min 100:0, 60–70 min 100:0, and 70–end min 8:92. The sample injection volume was 20 μL, the UV detector was set at 280 nm, and the flow rate was 1 mL/min. Rutin (y = 11361x + 426.15) and quercetin (y = 24495x + 52.046) were purchased from Sigma-Aldrich Co., and the calibration curves of standard materials all showed an R2 value of 0.999 or higher. All samples were analyzed in triplicate.

### 3.5. Measurement of ABTS and DPPH Radical Acavenging Activity

The ABTS radical scavenging activities of the SCW-treated rutin and its solvent fractions were measured using the method by Choi et al. [33]. ABTS radical cations were obtained by adding 7 mM ABTS to 2.45 mM potassium sulfate solution and stirring the mixture for 12–16 h at 25 °C, in the dark. The absorbance of the resulted solution was 1.5. One milliliter of the diluted ABTS radical cation solution was adjusted to a concentration of 0.5 mg/mL and was added to the extract or to 50 µL of distilled water (blank). After setting for 1 h, the absorbance was measured at 735 nm using a spectrophotometer. The electron-donating ability of the ABTS was expressed as mg of ascorbic acid per 1 g of the sample, measured by the difference in absorbance between the control and added groups. The DPPH radical scavenging activities of the SCW-treated rutin and its solvent fractions were analyzed using the method by Hwang et al. [34], where 0.8 mL of 0.2 mM DPPH (Sigma-Aldrich Co.) was added to 0.2 mL of the extract (0.1 mg/mL) or distilled water (blank), and the mixture was left at 25 °C for 30 min. The absorbance was analyzed at 520 nm. The electron-donating ability of the DPPH was expressed as mg ascorbic acid per 1 g of the sample, analyzed by the difference in absorbance between the control and added groups.

### 3.6. Measurement of Reducing Power

The reducing power was measured using method by Mau et al. [35], where 250 μL of 1% potassium ferricyanide [K_3_Fe(CN)_6_] and 250 μL of 0.2 M sodium phosphate buffer (pH 6.6) were mixed with 250 μL of extract and incubated at 50 °C for 20 min. Then, 1% trichloroacetic acid (CCl_3_COOH, *w*/*v*) was added, and the resulted solution was centrifuged at 1000 rpm for 10 min. Next, 500 μL of distilled water were mixed with 500 μL of the supernatant together with 100 μL of 0.1% ferric chloride (FeCl_3_∙6H_2_O), and the absorbance of the solution was analyzed at 700 nm.

### 3.7. Measurement of Tyrosinase Inhibitory Activity

To determine the skin-whitening effect, the inhibited tyrosinase activity was analyzed using the method by Yagi et al. [36]. In a test tube containing 0.2 mL of 10 mM L-3,4-dihydroxyphenylalanine (DOPA) dissolved in 0.5 mL of 1/15 M sodium phosphate buffer (pH 6.8) and 0.1 mL of sample solution, 0.2 mL of 110 Unit/mL mushroom tyrosinase was added. The mixture was incubated at 25 °C for 2 min, and the absorbance of the generated DOPA chrome was measured at 475 nm using a microplate reader. The inhibition of tyrosinase activity was indicated by a decrease in the absorbance of the group with and without the sample solution.

### 3.8. Measurement of Cytotoxicity

The cytotoxicity was analyzed using the method by Carmichael et al. [37], where B16F10 cells were seeded at 1 × 10^4^ cells/well in a 96 well plate, and CCD-986sk cells were seeded at 5 × 10^3^ cells/well in a 96 well plate and cultured for 24 h. After inoculation, the sample was diluted and incubated for 24 h, followed by the addition of 40 µL of MTT solution (2.5 mg/mL) and incubation for less than 4 h. After removing the culture medium, 100 µL of DMSO was added to the formazan, generated by the MTT reduction reaction, and incubated at 25 °C for 10 min. The absorbance was measured at 540 nm using an ELISA reader. The cytotoxicity was expressed as a percentage by comparing the sample’s absorbance with that of the control group (sample without any addition).

### 3.9. Measurement of Melanin Synthesized on Inhibition

The production of melanin was measured using the slightly modified method of Hosoi et al. [38]. B16F10 cells were aliquoted into a 100 mm tissue culture plate at 1 × 10^6^ cells/well, cultured for 24 h, and treated with 1 µg/mL of the stimulant α-MSH for 2 h. The sample was then diluted, inoculated, and incubated for 24 h. α-MSH was administered to the single treatment group and a sample section, whereas a section was left without α-MSH treatment and was used as the control group. The cultured plate was washed with PBS twice, and 80 µL of lysis buffer (67 mM sodium phosphate buffer, 1% Triton X-100, and 0.1 mM phenylmethyl sulfurylfluoride) were added. The resulting pellet was harvested and centrifuged at 13,200 rpm for 20 min. The supernatant was collected, and the protein contents were measured using a BSA kit and a standard curve. After centrifugation, the obtained pellet was dissolved in 10% DMSO mixed with 1 N NaOH solution. After dissolution, the absorbance was measured at 405 nm after the solution was incubated at 90 °C for 1 h using an ELISA reader. The melanin production was calculated and expressed as the percentage of the sample treatment group relative to the control group.

### 3.10. Measurement of Protein Expression Level Using Western Blot

To evaluate the protein expression of tyrosinase, microphthalmia transcription factor (MITF), TRP-1, and TRP-2 factors (whitening expression factors), B16F10 cells were seeded in a 100 mm tissue culture dish at 1 × 10^6^ cells/well and cultured for 24 h, after which, they were treated with 1 µg/mL α-MSH for 2 h. Extracts of 2.5, 5, and 10 µg/mL were incubated for 24 h, after which the culture medium was removed, and treatment with phosphate-buffered saline (PBS) followed. After washing twice, 100 µL of protease and phosphatase single-use inhibitor cocktail 100X was added to 10 mL of RIPA buffer, lysed with lysis buffer, and centrifuged at 13,200 rpm at 4 °C for 20 min. The protein content of the supernatant was quantified using a BCA protein assay kit, and 20 µL of protein was separated by electrophoresis using 10% SDS-PAGE. The separated protein was transferred to a polyvinylidekslne fluoride (PVDF) membrane and incubated by blocking for 1 h with 5% skim milk. Primary antibodies against tyrosinase, MITF, TRP-1, and TRP-2 were diluted 1:1000 in 3% skim milk and incubated overnight. After three 10-min washings with tris-buffered saline and Tween 20 (TBST), the secondary anti-mouse antibody was diluted 1:2000, 1:5000, and 1:10,000 in 3% skim milk and reacted at room temperature for 2 h. The washing process was repeated 3 times by stirring with TBST for 10 min. After adding the ECL solution, the band was confirmed and quantified using the ChemiDoc Imaging System.

### 3.11. Statistical Analysis

The mean and standard deviation of each measurement group were calculated using the Statistical Package for the Social Science program (SPSS, Ver. 18.0; SPSS Inc., Chicago, IL, USA), and the difference between the means of the measured values was independent. A one-way ANOVA and sample *t*-test (Student’s *t*-test) were performed to determine any differences between the treatment conditions.

## 4. Conclusions

This study analyzed the functional components, antioxidant activity, and skin-whitening effect of sub- and SCW-treated rutin. The temperature/pressure conditions tested were 200 °C/15 bar, 300 °C/100 bar, and 400 °C/250 bar. The highest quercetin content (381.39 mg/g) was observed in 200 °C/15 bar-treated rutin, which decreased as the temperature and pressure increased. Although the quercetin content was lower at 300 °C/100 bar, and 400 °C/250 bar than at 200 °C/15 bar, the ABTS and DPPH assay showed the highest values at 300 °C/100 bar. The tyrosinase inhibitory activity was also the highest at 300 °C/100 bar. After solvent fractionation, the ABTS and DPPH radical scavenging activities, reducing power, and tyrosinase inhibitory activity were the highest in the ethyl acetate fraction. The ethyl acetate fraction showed 14.91% inhibitory activity on melanin synthesis at a concentration of 10 µg/mL. The inhibition rates of MITF, tyrosinase, TRP-1, and TRP-2 in the ethyl acetate fractions were 14.05%, 72%, 93.05%, and 53.44%, respectively, at a concentration of 10 µg/mL. In summary, when rutin is treated with SCW, various reactions that can affect the structure of components, including decomposition reactions, occur. As a result, rutin is decomposed or synthesized to create new substances, which can cause changes in various physiological activities. Therefore, SCW treatment can be utilized to develop cosmetic materials and functional food with enhanced physiological activity. The results of this study also indicated that SCW-treated rutin can be used as a skin-whitening cosmetic material. Future studies are needed to investigate the mechanisms for increasing the antioxidant and skin-whitening activity of SCW-treated rutin and identify the active compounds.

## Figures and Tables

**Table 1:** Rutin, quercetin, ABTS, and DPPH radical scavenging activity and reducing power of rutin treated by different pressure and temperature.

Sample	Rutin (mg/g)	Quercetin (mg/g)	ABTS Radical Scavenging (mg AAE/g)	DPPH Radical Scavenging (mg AAE/g)	Reducing Power
					(Spectrophotometer 700 nm)
Rutin	1000	N.D.	465.97 ± 29.73d ^4^	656.08 ± 5.02d	0.31 ± 0.00d
200 °C 15 bar	N.D. ^1^	381.39 ± 1.52a ^2,3^	787.96 ± 42.13c	674.39 ± 5.40c	0.41 ± 0.00c
300 °C 100 bar	N.D.	218.12 ± 12.41b	1193.72 ± 6.93a	728.73 ± 1.34a	0.65 ± 0.00a
400 °C 250 bar	N.D.	1.40 ± 0.06c	945.03 ± 14.58b	694.88 ± 4.69b	0.47 ± 0.00b

^1^ Not detected; ^2^ mg ascorbic acid equivalent (AAE) per g. ^3^ Each value expressed as the mean ± standard deviation (*n* = 3). ^4^ Different letters in the same items indicate a significant difference by Duncan’s range test (*p* < 0.05).

**Table 2:** ABTS and DPPH radical scavenging activity and reducing power of solvent fraction for rutin treated by 300 °C and 100 bar.

Sample	Rutin (mg/g)	Quercetin (mg/g)	ABTS Radical Scavenging (mg AAE/g) ^4^	DPPH Radical Scavenging (mg AAE/g)	Reducing Power
					(700 nm)
Hexane	N.D. ^1^	0.80 ± 0.03 c ^2,3^	845.55 ± 2.62 b	542.17 ± 24.81 c	0.47 ± 0.00 b
Chloroform	N.D.	20.98 ± 0.30 b	815.88 ± 27.25 b	691.13 ± 8.27 b	0.48 ± 0.02 b
Ethyl acetate	N.D.	248.48 ± 6.63 a	1358.64 ± 9.07 a	1410.73 ± 10.04 a	0.77 ± 0.00 a
Butanol	N.D.	N.D.	161.43 ± 26.87 c	197.15 ± 9.86 d	0.14 ± 0.00 c
Water	N.D.	N.D.	N.D.	N.D.	N.D.

^1^ Not detected. ^2^ Each value expressed as the mean ± standard deviation (*n* = 3). ^3^ Different letters in the same items indicate a significant difference by Duncan’s range test (*p* < 0.05). ^4^ mg ascorbic acid equivalent (AAE).

## Data Availability

Not applicable.
